# Supplementary material for: Effects of physical, psychosocial and dual-career loads on injuries and illnesses among elite handball players: protocol of prospective cohort study
Source: BMJ Open. 2023 Mar 2;13(3):e069104. doi: 10.1136/bmjopen-2022-069104 (PMC9990604; doi:10.1136/bmjopen-2022-069104)
Supplement: Author's manuscript [file bmjopen-2022-069104.draft_revisions.pdf]

BMJ Open is committed to open peer review. As part of this commitment we make the peer review history of every article we publish publicly available.

When an article is published we post the peer reviewers' comments and the authors' responses online. We also post the versions of the paper that were used during peer review. These are the versions that the peer review comments apply to.

The versions of the paper that follow are the versions that were submitted during the peer review process. They are not the versions of record or the final published versions. They should not be cited or distributed as the published version of this manuscript.

BMJ Open is an open access journal and the full, final, typeset and author-corrected version of record of the manuscript is available on our site with no access controls, subscription charges or pay-per-view fees (http://bmjopen.bmj.com).

If you have any questions on BMJ Open's open peer review process please email info.bmjopen@bmj.com

BMJ Open

Effects of physical, psychosocial and dual-career loads on injuries and illnesses among elite handball players: protocol of the study

Journal:	*BMJ Open*
Manuscript ID	bmjopen-2022-069104
Article Type:	Protocol
Date Submitted by the Author:	13-Oct-2022
Complete List of Authors:	Drole, Kristina; University of Ljubljana<br>Paravlic, Armin; University of Ljubljana; Masaryk University<br>Steffen, Kathrin; Norwegian School of Sport Sciences<br>Doupona, Mojca; University of Ljubljana
Keywords:	SPORTS MEDICINE, Health policy < HEALTH SERVICES ADMINISTRATION & MANAGEMENT, IMMUNOLOGY, Biochemistry < NATURAL SCIENCE DISCIPLINES

SCHOLARONE™
Manuscripts

Proposed journal: BMJ Open

Title: Effects of physical, psychosocial and dual-career loads on injuries and illnesses among elite handball players: protocol of the study

Authors: Kristina Drole[1], Armin Paravlic[1,2,3], Kathrin Steffen[4], Mojca Doupona[1]

**Corresponding author:*

Kristina Drole
Faculty of Sport, University of Ljubljana
Gortanova 22, 1000 Ljubljana, Slovenia
E-mail: Kristina.drole@fsp.uni-lj.si

[1] Faculty of Sport, University of Ljubljana, Ljubljana, Slovenia
[2] Science and Research Centre Koper, Institute for Kinesiology Research, Koper, Slovenia
[3] Faculty of Sports Studies, Masaryk University, Brno, Czechia
[4] Oslo Sports Trauma Research Center, Norwegian School of Sport Sciences, Oslo, Norway

ORCID:
Kristina Drole: 0000-0002-8403-3154
Armin Paravlic: 0000-0002-7748-8097
Kathrin Steffen: 0000-0002-6238-608X
Mojca Doupona: 0000-0002-6715-0391

Word count: 2070

For peer review only - http://bmjopen.bmj.com/site/about/guidelines.xhtml

Abstract

Introduction

Health problems in sport cause a major burden on several pillars: sport clubs, health and insurance system and mostly the individual athlete. There is limited knowledge in supporting dual-career athletes based on evidence-based research in injury/illness prevention, load and stress management. The main goal of this research approach is to determine how specific physical, psychosocial and dual-career loads affect the occurrence of injuries and illnesses in elite handball players and how much of a variation in the athlete's load leads to an occurrence of an injury/illness. A secondary aim is to determine the association between objective and subjective measures of stress, as well examine the benefits of certain biomarkers to monitor stress, load and injury/illness occurrence in athletes.

Methods and analysis

This prospective cohort study, as part of a PhD-project, will be carried out on 200 elite handball players of 1st men handball league in Slovenia during an entire handball season, lasting from July 2022 to June 2023. Primary outcomes, including health problems, loads and stress, will be assessed weekly on a player level. Other player-related outcomes will include anthropometry, life event survey and blood biomarkers (cortisol, free testosterone and Ig-A), which will be taken three to five times across the observation period according to the players´ training cycle.

Ethics and dissemination

The project was approved by the National Medical Ethics Committee of Slovenia (number: 0120-109/2022/3) and will be conducted in compliance with the most recent version of Helsinki Declaration. The study results will be published as peer-reviewed articles, congress presentations and as a Doctoral thesis. The results will not only be of importance for the medical and sports community for development of new injury prevention and rehabilitation strategies, but also for structuring the correct policy recommendations for athletes' general health.

Registration details

Trial registration number: NCT0547129

Keywords: monitoring, athletic injuries, health problems, biomarkers, load management

For peer review only

For peer review only - http://bmjopen.bmj.com/site/about/guidelines.xhtml

Article Summary

Strengths and limitations of this study

- This longitudinal study will investigate the associations between different types of load and injury/illness occurrence in elite handball players
- The study will implement holistic monitoring of athlete's health with objective and subjective measures
- The association between objective and subjective measures of stress in athletes will be investigated
- The study focuses exclusively on elite male handball players, which can be seen both as a strength and also as a limitation when it comes to generalizability
- The work has direct implications for sports/clinical practice; periodical athlete monitoring could be beneficial in terms of preventing health issues, lowering health system costs, supporting dual career of athletes, structuring sound policies and enhancing both sports and academic performance

For peer review only

For peer review only - http://bmjopen.bmj.com/site/about/guidelines.xhtml

Introduction

Although regular moderate exercise is known to have positive health benefits[1]; from preventing a number of non-communicable diseases to improving cardio-respiratory fitness and consequently decreasing cardiovascular morbidity and mortality.[1–3] Top-level sports on the other hand, are associated with an increased risk for illness and injury.[4,5] There has been a number of studies[6–12] showing a high prevalence of health issues that include injuries and illnesses among elite athletes, ranging from 20% in athletes of all ages[4] to 45% in youth athletes.[5] The injuries and illnesses are not only limiting athletes for the time being[13], but also represent a major burden for sport clubs, health and insurance system and can as well be the reason for sports career termination.[14–18]

Athlete's stressors

Sports and non-sports loads impose stress on athletes, leading their physical and psychological well-being from homeostasis to acute fatigue, over-reaching, overtraining, subclinical immune changes, clinical symptoms, illness/injury and possibly death.[19] Research points out that inadequate load management is an important independent risk factor for the development of acute illness and overtraining syndrome.[9,19,20] However, it is not yet possible to quantify how much an increase in training load leads to an increased risk for a given illness[19] or injury.[21] Furthermore, the literature states that there is a risk of illness when an athlete is exposed to other lifestyle stressors that affect the immune system, including sleep deprivation or severe psychosocial stress[22], such as major life events.[23] A special group of athletes, that might be at higher risk for health problems are dual-career (DC) athletes. They are posed to the challenge of combining their education/work with elite sport, suggesting their overall weekly load is higher compared to non-athlete students and athletes who are not in the education process. It was found that the highest levels of stress and illness incidence in student-athletes arise during the academically stressful periods.[24,25] Thus, the relationship between stress and physical health is especially relevant for dual-career athletes, who often face multiple physical and psychosocial stressors (such as transitioning between schools/campuses and home life, frequent travel, academic load and sports load).[26–28]

Health monitoring and load management

One potential strategy for injury and illness prevention in elite sport is monitoring of external and internal loads. Monitoring an athlete's workload has a number of potential benefits, including interpreting performance changes, increasing understanding of exercise responses, detecting fatigue and monitoring recovery needs, optimization of the training programme and competition calendar. It also serves for providing adequate workload levels to reduce the risk of injury and illness[29,30] and guide rehabilitation programmes. External and internal loads can be assessed with subjective and objective measures. External load measurement typically involves quantifying an athlete's training or competition loads by reporting training hours, running length, amount of weight lifted, number of games played and other external factors such as life events, everyday problems and/or travel. On the other hand, internal load is measured by assessing the internal biological, physiological, and psychological response to an external load[31], such as heart rate or rate of perceived exertion. A recent systematic review of internal stress monitoring has identified subjective reporting to be more sensitive and consistent than objective measurements in determining acute and chronic changes in an athlete's well-being.[32]

For peer review only - http://bmjopen.bmj.com/site/about/guidelines.xhtml
For peer review only

Biomarkers

Biomarker discovery and validation is a critical aim of the medical and scientific community.[33] Biomarkers can be used as an objective assessment tool for health, performance, recovery and talent identification in elite sport. Among the most critical hormones for athletes seem to be cortisol, testosterone, dehydroepiandrosterone (DHEA) and immunoglobulin-A (Ig-A). However, T:C ratio is considered to be more sensitive to training stress than either measure alone[33], as several studies report poor performance outcomes and suboptimal training adaptations in athletes with a low T:C ratio.[34,35] However, to the best of the authors knowledge, no previous work has examined the use of biomarkers for injury prevention, recovery after an injury or identification of individuals at increased risk.

Given the all above, there is a need for better health surveillance, load monitoring and support services with an emphasis on injury/illness prevention and rehabilitation.[36] As DC athletes have a wider variety of loads that pose greater psychophysiological strain to them, they could also be at an increased risk for injury/illness. Therefore, more comprehensive monitoring of potential risk factors in this specific population is warranted. It is not yet known, but important to determine how much of overall load can an athlete withstand before injury/illness occurs. Thus, the primary aim of this study is to determine how specific physical, psychosocial and dual-career loads affect the occurrence of injuries and illnesses in elite handball players and how much of a variation in the athlete's load leads to an occurrence of an injury/illness. A secondary aim is to determine the association between objective and subjective measures of stress, as well as examine the benefits of certain biomarkers to monitor stress, load and injury/illness occurrence in athletes.

Methods and analysis

Study design

The study is designed as prospective cohort study. All measurements will be conducted over a single handball season, lasting from July 2022 to June 2023 (a total of 46 weeks), with weekly reporting (Table 1) and three to five biomarker sampling timepoints (Figure 1). We used the SPIRIT checklist when writing our report. [37]

Participants and sample size

A power analysis was a priory determined using the G*Power software.[38] Based on previous studies with similar design we expected to find nearly twice as high odds of injury occurrence in athletes during a period of high academic stress compared to low academic stress (odds ratio [OR] = 1.78; effect size [ES] = 0.14)[25] with power of 0.90 and α = 0.05, two-tailed, which calculated an initial sample size of 134 participants. As in sports practice, the athlete drop-out is to be expected (due to travelling, international transfer to another club/state etc.); we adjusted the originally calculated sample size by following formula: N1 = n/(1-d)[39], where N1 is adjusted sample size, n is the sample size required as per the proposed formula (N = 134), and d is the drop-out rate (d = 0.10). This resulted in total sample size of 149 athletes. Inclusion criteria will be as follows: DC and non-DC registered male handball players who are competing in the 1st Slovenian handball league and are above 18 years of age.

For peer review only - http://bmjopen.bmj.com/site/about/guidelines.xhtml

Assessment and Outcomes

*** Insert Figure 1 "Assessment plan" approximately here ***

Oslo Sports Trauma Research Centre Questionnaire on Health Problems (OSTRC-H2)

The OSTRC questionnaire is a tool to capture and monitor health problems longitudinally in athletes. It consists of 18 questions, which are answered for the past seven days. The first four questions are to determine the severity of the health problem, while the rest are to further classify the health problem, such as type and anatomical location of the problem.[40] As per previously established protocol, the athletes in our study will self-report their health status weekly with the help of a physiotherapist, while using the OSTRC questionnaire.[40] The athletes with reported health problem will be contacted by a medical professional to clinically verify the reported health case.

Life Events Survey for Collegiate Athletes Questionnaire (LESCA)

At the baseline and final measurements, the athletes will be asked to complete LESCA questionnaire[41] in order to get more contextualized view of the life events that were happening in the past 12 months. LESCA is a 69-item questionnaire where athletes rate how each life event affected their lives in the past 12 months on a -4 to 4-point scale (-4, extremely negative to +4, extremely positive). The events include personal, academic-related and sports-related events. From summing the rated life events, two life stress scores, negative (NEG) and positive (POS) are obtained, while a total life stress score can as well be obtained by summing the NEG and POS scores together.[41]

Weekly load and stress Questionnaire

The teams will weekly report training and competition load for each athlete, while academic (educational activities, including lectures, exams and studying) and work load (a part-time or full-time job besides playing sports) for the past seven days will be reported by the athletes themselves. Competition load will be reported in minutes for each game played in the past week, while training, academic and workload will be reported in hours. The athletes will additionally report average hours slept per day over the past week and rate their perceived overall stress of that week on the scale 1-10.

Blood biomarkers

There will be three to five measurement timepoints for biomarker sampling, according to the training (preparatory period, competitive period, off-season period) cycle. The athletes will be tested under similar conditions every time, after a rest day (refrainment from intensive training the day before blood collection), between 7-10 a.m. after a light breakfast.

Table 1. Questionnaires and tests.

Test/Questionnaire	Outcome	Purpose
**SDSCQ**	Age, sex, level of education, sport, years of training, player position, past injuries	General information about the athlete

For peer review only - http://bmjopen.bmj.com/site/about/guidelines.xhtml

**Anthropometry**	Body height (cm), body weight (kg)	Measure of basic anthropometric characteristics of an athlete
**LESCA**	Major life events score	Measure of life stress
**OSTRC**	Weekly health problems (acute and overuse injuries, illnesses), measured as prevalence and incidence and severity as OSTRC-severity score and time-loss days	Identifying health problems
**Weekly load and stress**		Monitoring of athlete's load
	Training load in hours	
	Competition load in minutes	
	Academic load in hours	
	Work load in hours	
	Average daily sleeping hours	
	Perceived stress on scale 1-10	
**Blood biomarkers**		Monitoring of stress/insufficient recovery and/or health problem risk
Cortisol	Cortisol values in nmol/L	
Free testosterone	Free testosterone values in pmol/L	
Ig-A	Ig-A values in g/L	
	Free testosterone: cortisol ratio	

SDSCQ, sociodemographic and sports-career related questionnaire; LESCA, Life Events Survey for Collegiate Athletes; OSTRC, Oslo Sports Trauma Research Center Questionnaire on Health Problems

Statistical analysis

Statistical analysis will be performed using standard statistical software (SPSS 27.0, IBM Corp, Armonk, NY, USA). Descriptive statistics will be used for all variables. The data will be analysed using a logistic regression model to properly account for the dependency induced by the repeated observations over time within each subject. Main effect of all dependent variables on injury/illness occurrence will be studied using repeated measures ANOVA with time (preparation period vs. competitive period vs. off-season period) as within factor, while group (Injured/ill vs. Healthy) as between factor. Where significant effects will be found for group, time effects or 2-way interactions, pairwise comparisons will be used to address significant differences for each variable independently. For comparison of the groups we will use independent t-test. The magnitude of effect for all dependent variables will be reported as partial eta squared (η^2). In addition, *Cohens' d* (ES) was used to assess magnitude of difference between groups. Furthermore, regression analysis will be used to assess predictive power of individual parameters to identify injury/illness occurrence. For the correlation of subjective measures of stress with the objective measures, bivariate correlation will be used together with Bland-Altmann (low/high stress normative values). Furthermore, ROC curve will be used for defining the cut-off values. Hypotheses will be accepted or rejected at $P \leq 0.05$.

Patient and public involvement

Patients and/or the public were not involved in the design and/or conduct, and/or reporting of this study. The project results will be presented to the participants in collaboration with the team staff (strength and conditioning coaches, physiotherapists, kinesiologists, etc.). Furthermore, the participants of the proposed study will be invited to share their experiences of their involvement in our project with elite

For peer review only - http://bmjopen.bmj.com/site/about/guidelines.xhtml

athletes playing other sports in the Republic of Slovenia in order to continue to implement "good practice" with future projects.

Ethics and dissemination

The project was approved by the National Medical Ethics Committee of Slovenia (number: 0120-109/2022/3) and registered on Clinical Trials.gov (registration number: NCT05471297). The study will be conducted in compliance with the most recent version of Helsinki Declaration of 1975 and the American College of Sports Medicine Policy Statement Regarding the Use of Human Subjects and Informed Consent. Any changes made to the study protocol will be submitted to the National Medical Ethics Committee for consideration and approval. Participants will be introduced to the aims and objectives of the study, noticed that participation in the study is voluntary and asked to sign an informed consent, collected by KD. Consent document also states that the participant is free to withdraw from the study without impact on their future status in the team and without having to provide the reason for their withdrawal. Data on each participant will be anonymised using only ID numbers. The study will be coordinated by KD, a Ph.D. student conducting her Ph.D. thesis on this topic. As such, the study results will be published as peer-reviewed articles, conference presentations and finally, as a Doctoral thesis.

Acknowledgements

The authors wish to thank Biolab laboratorijska diagnostika d.o.o. and University Medical Centre Ljubljana, Clinical Institute of Clinical Chemistry and Biochemistry for supporting the upcoming blood biomarker analysis.

Author contributions

KD contributed to the idea, conceptualization and drafted the manuscript; KD and AP contributed to design and planned analysis of the study. All authors critically revised the manuscript and gave their final approval.

Funding

The work is supported by a research fellowship grant (no. 393/2020) received by KD from the Public Research Agency of the Republic of Slovenia (ARRS). The research took place within the KINESIOLOGY OF MONOSTRUCTURAL, POLYSTRUCTURAL AND CONVENTIONAL SPORTS research program, code: P5-0147, financed by the Public Research Agency of the Republic of Slovenia. The funding agency has no impact on data collection, analysis or interpretation of the study results.

Competing interests

The authors report no competing interest within the submitted work.

Data statement

Data will be provided by the corresponding author upon a reasonable request.

For peer review only - http://bmjopen.bmj.com/site/about/guidelines.xhtml

References

1 Ruegsegger GN, Booth FW. Health Benefits of Exercise. *Cold Spring Harb Perspect Med* 2018;**8**. doi:10.1101/cshperspect.a029694

2 Warburton DER, Nicol CW, Bredin SSD. Health benefits of physical activity: the evidence. *C Can Med Assoc J = J l'Association medicale Can* 2006;**174**:801–9. doi:10.1503/cmaj.051351

3 Warburton DER, Bredin SSD. Health benefits of physical activity: a systematic review of current systematic reviews. *Curr Opin Cardiol* 2017;**32**:541–56. doi:10.1097/HCO.0000000000000437

4 Pluim BM, Fuller CW, Batt ME, *et al.* Consensus statement on epidemiological studies of medical conditions in tennis, April 2009. *Clin J Sport Med Off J Can Acad Sport Med* 2009;**19**:445–50. doi:10.1097/JSM.0b013e3181be35e5

5 Moseid CH, Myklebust G, Fagerland MW, *et al.* The prevalence and severity of health problems in youth elite sports: A 6-month prospective cohort study of 320 athletes. *Scand J Med Sci Sports* 2018;**28**:1412–23. doi:10.1111/sms.13047

6 Steffen K, Moseid CH, Engebretsen L, *et al.* Sports injuries and illnesses in the Lillehammer 2016 Youth Olympic Winter Games. *Br J Sports Med* 2017;**51**:29–35. doi:10.1136/bjsports-2016-096977

7 Steffen K, Soligard T, Mountjoy M, *et al.* How do the new Olympic sports compare with the traditional Olympic sports? Injury and illness at the 2018 Youth Olympic Summer Games in Buenos Aires, Argentina. *Br J Sports Med* 2020;**54**:168–75. doi:10.1136/bjsports-2019-101040

8 Engebretsen L, Soligard T, Steffen K, *et al.* Sports injuries and illnesses during the London Summer Olympic Games 2012. *Br J Sports Med* 2013;**47**:407–14.

9 Soligard T, Steffen K, Palmer-Green D, *et al.* Sports injuries and illnesses in the Sochi 2014 Olympic Winter Games. *Br J Sports Med* 2015;**49**:441–7. doi:10.1136/bjsports-2014-094538

10 Steffen K, Clarsen B, Gjelsvik H, *et al.* Illness and injury among Norwegian Para athletes over five consecutive Paralympic Summer and Winter Games cycles: prevailing high illness burden on the road from 2012 to 2020. *Br J Sports Med* 2022;**56**:204–12. doi:10.1136/bjsports-2021-104489

11 Theron N, Schwellnus M, Derman W, *et al.* Illness and injuries in elite football players--a prospective cohort study during the FIFA Confederations Cup 2009. *Clin J Sport Med Off J Can Acad Sport Med* 2013;**23**:379–83. doi:10.1097/JSM.0b013e31828b0a10

12 Rosen P von, Heijne A, Frohm A, *et al.* High Injury Burden in Elite Adolescent Athletes: A 52-Week Prospective Study. *J Athl Train (Allen Press* 2018;**53**:262–70.

13 Caine DJ, Golightly YM. Osteoarthritis as an outcome of paediatric sport: an epidemiological perspective. *Br J Sports Med* 2011;**45**:298–303. doi:10.1136/bjsm.2010.081984

14 Drawer S, Fuller CW. Propensity for osteoarthritis and lower limb joint pain in retired professional soccer players. *Br J Sports Med* 2001;**35**:402–8. doi:10.1136/bjsm.35.6.402

15 Palmer D, Cooper DJ, Emery C, *et al.* Self-reported sports injuries and later-life health status in 3357 retired Olympians from 131 countries: A cross-sectional survey among those competing in the games between London 1948 and PyeongChang 2018. *Br J Sports Med* 2021;**55**:46–53. doi:10.1136/bjsports-2019-101772

16 Drole K. *ZDRAVSTVENI STATUS IN POŠKODBE BIVŠIH VRHUNSKIH ŠPORTNIKOV*. 2020.

17 Leena R, Kettunen J, Kujala U, *et al.* Sport injuries as the main cause of sport career

For peer review only - http://bmjopen.bmj.com/site/about/guidelines.xhtml

termination among Finnish top-level athletes. *Eur J Sport Sci* 2012;**12**:274–82. doi:10.1080/17461391.2011.566365

18 Kettunen JA, Kujala UM, Kaprio J, *et al.* Lower-limb function among former elite male athletes. *Am J Sports Med* 2001;**29**:2–8. doi:10.1177/03635465010290010801

19 Schwellnus M, Soligard T, Alonso J-M, *et al.* How much is too much? (Part 2) International Olympic Committee consensus statement on load in sport and risk of illness. *Br J Sports Med* 2016;**50**:1043–52. doi:10.1136/bjsports-2016-096572

20 Gleeson M, Pyne DB. Respiratory inflammation and infections in high-performance athletes. *Immunol Cell Biol* 2016;**94**:124–31. doi:10.1038/icb.2015.100

21 Dalen-Lorentsen T. *Training load and health problems in football –More complex than we first thought?* 2021.

22 NIEMAN DC. Exercise effects on systemic immunity. *Immunol Cell Biol* 2000;**78**:496–501. doi:https://doi.org/10.1111/j.1440-1711.2000.t01-5-.x

23 Smith RE, Smoll FL, Ptacek JT. Conjunctive moderator variables in vulnerability and resiliency research: Life stress, social support and coping skills, and adolescent sport injuries. J. Pers. Soc. Psychol. 1990;**58**:360–70. doi:10.1037/0022-3514.58.2.360

24 Hamlin MJ, Wilkes D, Elliot CA, *et al.* Monitoring Training Loads and Perceived Stress in Young Elite University Athletes. *Front Physiol* 2019;**10**:34. doi:10.3389/fphys.2019.00034

25 Mann JB, Bryant KR, Johnstone B, *et al.* Effect of Physical and Academic Stress on Illness and Injury in Division 1 College Football Players. *J strength Cond Res* 2016;**30**:20–5. doi:10.1519/JSC.0000000000001055

26 Condello G, Capranica L, Doupona M, *et al.* Dual-career through the elite university student-athletes' lenses: The international FISU-EAS survey. *PLoS One* 2019;**14**:e0223278.https://doi.org/10.1371/journal.pone.0223278

27 Fuchs PX, Doupona M, Varga K, *et al.* Multi-national perceptions on challenges, opportunities, and support structures for Dual Career migrations in European student-athletes. *PLoS One* 2021;**16**:e0253333. doi:10.1371/journal.pone.0253333

28 Cosh SM, Tully PJ. Stressors, coping, and support mechanisms for student athletes combining elite sport and tertiary education: implications for practice. *IEEE Trans Signal Process* 2015;**29**:120–33.

29 Halson SL. Monitoring training load to understand fatigue in athletes. *Sports Med* 2014;**44 Suppl 2**:S139-47. doi:10.1007/s40279-014-0253-z

30 Foster C. Monitoring training in athletes with reference to overtraining syndrome. *Med Sci Sports Exerc* 1998;**30**:1164–8. doi:10.1097/00005768-199807000-00023

31 Borresen J, Lambert MI. The quantification of training load, the training response and the effect on performance. *Sports Med* 2009;**39**:779–95. doi:10.2165/11317780-000000000-00000

32 Saw AE, Main LC, Gastin PB. Monitoring the athlete training response: Subjective self-reported measures trump commonly used objective measures: A systematic review. *Br J Sports Med* 2016;**50**:281–91. doi:10.1136/bjsports-2015-094758

33 Lee EC, Fragala MS, Kavouras SA, *et al.* Biomarkers in Sports and Exercise: Tracking Health, Performance, and Recovery in Athletes. *J strength Cond Res* 2017;**31**:2920–37. doi:10.1519/JSC.0000000000002122

34 Kraemer WJ, French DN, Paxton NJ, *et al.* Changes in exercise performance and hormonal concentrations over a big ten soccer season in starters and nonstarters. *J strength Cond Res*

For peer review only

For peer review only - http://bmjopen.bmj.com/site/about/guidelines.xhtml

2004;**18**:121–8. doi:10.1519/1533-4287(2004)018<0121:ciepah>2.0.co;2

35 Chicharro JL, López-Mojares LM, Lucía A, *et al.* Overtraining parameters in special military units. *Aviat Space Environ Med* 1998;**69**:562–8.

36 Drole K, Paravlic AH. Interventions for increasing return to sport rates after an anterior cruciate ligament reconstruction surgery: A systematic review. Front. Psychol. 2022;**13**:939209. doi:10.3389/fpsyg.2022.939209

37 Chan A-W, Tetzlaff JM, Gøtzsche PC, *et al.* SPIRIT 2013 explanation and elaboration: guidance for protocols of clinical trials. *BMJ* 2013;**346**:e7586. doi:10.1136/bmj.e7586

38 Faul F, Erdfelder E, Lang A-G, *et al.* G*Power 3: a flexible statistical power analysis program for the social, behavioral, and biomedical sciences. *Behav Res Methods* 2007;**39**:175–91. doi:10.3758/bf03193146

39 Sakpal TV. Sample size estimation in clinical trial. *Perspect Clin Res* 2010;**1**:67–9.

40 Clarsen B, Bahr R, Myklebust G, *et al.* Improved reporting of overuse injuries and health problems in sport: an update of the Oslo Sport Trauma Research Center questionnaires. *Br J Sports Med* 2020;**54**:390–6. doi:10.1136/bjsports-2019-101337

41 Petrie TA. Psychosocial antecedents of athletic injury: the effects of life stress and social support on female collegiate gymnasts. *Behav Med* 1992;**18**:127–38. doi:10.1080/08964289.1992.9936963

For peer review only

For peer review only - http://bmjopen.bmj.com/site/about/guidelines.xhtml

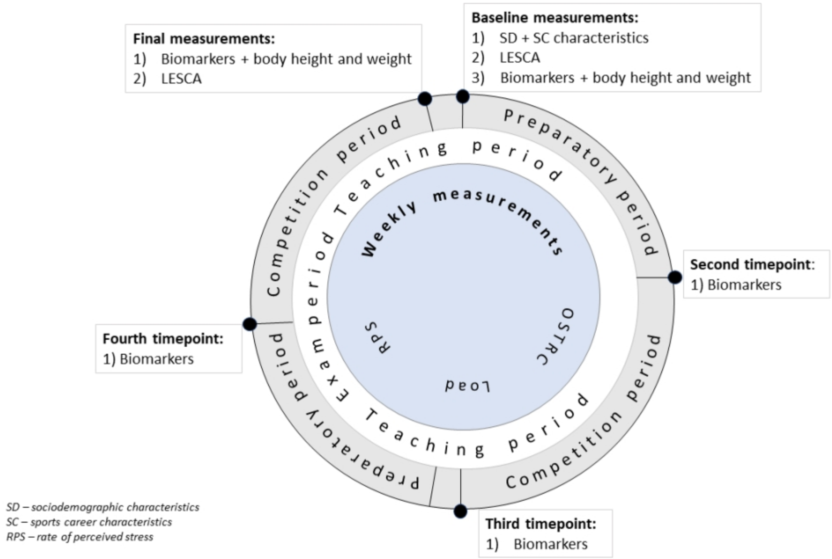

338x190mm (96 x 96 DPI)

Reporting checklist for protocol of a clinical trial.

Based on the SPIRIT guidelines.

Instructions to authors

Complete this checklist by entering the page numbers from your manuscript where readers will find each of the items listed below.

Your article may not currently address all the items on the checklist. Please modify your text to include the missing information. If you are certain that an item does not apply, please write "n/a" and provide a short explanation.

Upload your completed checklist as an extra file when you submit to a journal.

In your methods section, say that you used the SPIRITreporting guidelines, and cite them as:

Chan A-W, Tetzlaff JM, Gøtzsche PC, Altman DG, Mann H, Berlin J, Dickersin K, Hróbjartsson A, Schulz KF, Parulekar WR, Krleža-Jerić K, Laupacis A, Moher D. SPIRIT 2013 Explanation and Elaboration: Guidance for protocols of clinical trials. BMJ. 2013;346:e7586

		Reporting Item	Page Number
**Administrative information**			
Title	#1	Descriptive title identifying the study design, population, interventions, and, if applicable, trial acronym	1
Trial registration	#2a	Trial identifier and registry name. If not yet registered, name of intended registry	2
Trial registration: data set	#2b	All items from the World Health Organization Trial Registration Data Set	2
Protocol version	#3	Date and version identifier	2
Funding	#4	Sources and types of financial, material, and other support	8
Roles and responsibilities: contributorship	#5a	Names, affiliations, and roles of protocol contributors	1

For peer review only

Roles and responsibilities: sponsor contact information	#5b	Name and contact information for the trial sponsor	NA
Roles and responsibilities: sponsor and funder	#5c	Role of study sponsor and funders, if any, in study design; collection, management, analysis, and interpretation of data; writing of the report; and the decision to submit the report for publication, including whether they will have ultimate authority over any of these activities	8
Roles and responsibilities: committees	#5d	Composition, roles, and responsibilities of the coordinating centre, steering committee, endpoint adjudication committee, data management team, and other individuals or groups overseeing the trial, if applicable (see Item 21a for data monitoring committee)	NA
**Introduction**			
Background and rationale	#6a	Description of research question and justification for undertaking the trial, including summary of relevant studies (published and unpublished) examining benefits and harms for each intervention	4-5
Background and rationale: choice of comparators	#6b	Explanation for choice of comparators	NA
Objectives	#7	Specific objectives or hypotheses	5
Trial design	#8	Description of trial design including type of trial (eg, parallel group, crossover, factorial, single group), allocation ratio, and framework (eg, superiority, equivalence, non-inferiority, exploratory)	5
**Methods: Participants, interventions, and outcomes**			
Study setting	#9	Description of study settings (eg, community clinic, academic hospital) and list of countries where data will be collected. Reference to where list of study sites can be obtained	5
Eligibility criteria	#10	Inclusion and exclusion criteria for participants. If applicable, eligibility criteria for study centres and individuals who will	5

For peer review only

		perform the interventions (eg, surgeons, psychotherapists)	
Interventions: description	#11a	Interventions for each group with sufficient detail to allow replication, including how and when they will be administered	5-7
Interventions: modifications	#11b	Criteria for discontinuing or modifying allocated interventions for a given trial participant (eg, drug dose change in response to harms, participant request, or improving / worsening disease)	5
Interventions: adherance	#11c	Strategies to improve adherence to intervention protocols, and any procedures for monitoring adherence (eg, drug tablet return; laboratory tests)	NA
Interventions: concomitant care	#11d	Relevant concomitant care and interventions that are permitted or prohibited during the trial	6
Outcomes	#12	Primary, secondary, and other outcomes, including the specific measurement variable (eg, systolic blood pressure), analysis metric (eg, change from baseline, final value, time to event), method of aggregation (eg, median, proportion), and time point for each outcome. Explanation of the clinical relevance of chosen efficacy and harm outcomes is strongly recommended	6-7
Participant timeline	#13	Time schedule of enrolment, interventions (including any run-ins and washouts), assessments, and visits for participants. A schematic diagram is highly recommended (see Figure)	6
Sample size	#14	Estimated number of participants needed to achieve study objectives and how it was determined, including clinical and statistical assumptions supporting any sample size calculations	5
Recruitment	#15	Strategies for achieving adequate participant enrolment to reach target sample size	5
**Methods: Assignment of interventions (for controlled trials)**			
Allocation: sequence generation	#16a	Method of generating the allocation sequence (eg, computer-generated random numbers), and list of any factors for stratification. To reduce predictability of a random sequence, details of any planned restriction (eg, blocking) should be provided in a separate document that is unavailable to those who enrol participants or assign interventions	NA

For peer review only

Allocation concealment mechanism	#16b	Mechanism of implementing the allocation sequence (eg, central telephone; sequentially numbered, opaque, sealed envelopes), describing any steps to conceal the sequence until interventions are assigned	NA
Allocation: implementation	#16c	Who will generate the allocation sequence, who will enrol participants, and who will assign participants to interventions	NA
Blinding (masking)	#17a	Who will be blinded after assignment to interventions (eg, trial participants, care providers, outcome assessors, data analysts), and how	NA
Blinding (masking): emergency unblinding	#17b	If blinded, circumstances under which unblinding is permissible, and procedure for revealing a participant's allocated intervention during the trial	NA
**Methods: Data collection, management, and analysis**			
Data collection plan	#18a	Plans for assessment and collection of outcome, baseline, and other trial data, including any related processes to promote data quality (eg, duplicate measurements, training of assessors) and a description of study instruments (eg, questionnaires, laboratory tests) along with their reliability and validity, if known. Reference to where data collection forms can be found, if not in the protocol	5-7
Data collection plan: retention	#18b	Plans to promote participant retention and complete follow-up, including list of any outcome data to be collected for participants who discontinue or deviate from intervention protocols	5-7
Data management	#19	Plans for data entry, coding, security, and storage, including any related processes to promote data quality (eg, double data entry; range checks for data values). Reference to where details of data management procedures can be found, if not in the protocol	7-8
Statistics: outcomes	#20a	Statistical methods for analysing primary and secondary outcomes. Reference to where other details of the statistical analysis plan can be found, if not in the protocol	7
Statistics: additional analyses	#20b	Methods for any additional analyses (eg, subgroup and adjusted analyses)	7

For peer review only

Statistics: analysis population and missing data	#20c	Definition of analysis population relating to protocol non-adherence (eg, as randomised analysis), and any statistical methods to handle missing data (eg, multiple imputation)	7
**Methods: Monitoring**			
Data monitoring: formal committee	#21a	Composition of data monitoring committee (DMC); summary of its role and reporting structure; statement of whether it is independent from the sponsor and competing interests; and reference to where further details about its charter can be found, if not in the protocol. Alternatively, an explanation of why a DMC is not needed	NA
Data monitoring: interim analysis	#21b	Description of any interim analyses and stopping guidelines, including who will have access to these interim results and make the final decision to terminate the trial	NA
Harms	#22	Plans for collecting, assessing, reporting, and managing solicited and spontaneously reported adverse events and other unintended effects of trial interventions or trial conduct	NA
Auditing	#23	Frequency and procedures for auditing trial conduct, if any, and whether the process will be independent from investigators and the sponsor	NA
**Ethics and dissemination**			
Research ethics approval	#24	Plans for seeking research ethics committee / institutional review board (REC / IRB) approval	7-8
Protocol amendments	#25	Plans for communicating important protocol modifications (eg, changes to eligibility criteria, outcomes, analyses) to relevant parties (eg, investigators, REC / IRBs, trial participants, trial registries, journals, regulators)	7-8
Consent or assent	#26a	Who will obtain informed consent or assent from potential trial participants or authorised surrogates, and how (see Item 32)	7-8
Consent or assent: ancillary studies	#26b	Additional consent provisions for collection and use of participant data and biological specimens in ancillary studies, if applicable	NA
Confidentiality	#27	How personal information about potential and enrolled participants will be collected, shared, and maintained in order to protect confidentiality before, during, and after the trial	7-8

For peer review only

Declaration of interests	#28	Financial and other competing interests for principal investigators for the overall trial and each study site	8
Data access	#29	Statement of who will have access to the final trial dataset, and disclosure of contractual agreements that limit such access for investigators	8
Ancillary and post trial care	#30	Provisions, if any, for ancillary and post-trial care, and for compensation to those who suffer harm from trial participation	NA
Dissemination policy: trial results	#31a	Plans for investigators and sponsor to communicate trial results to participants, healthcare professionals, the public, and other relevant groups (eg, via publication, reporting in results databases, or other data sharing arrangements), including any publication restrictions	7-8
Dissemination policy: authorship	#31b	Authorship eligibility guidelines and any intended use of professional writers	8
Dissemination policy: reproducible research	#31c	Plans, if any, for granting public access to the full protocol, participant-level dataset, and statistical code	8
**Appendices**			
Informed consent materials	#32	Model consent form and other related documentation given to participants and authorised surrogates	11
Biological specimens	#33	Plans for collection, laboratory evaluation, and storage of biological specimens for genetic or molecular analysis in the current trial and for future use in ancillary studies, if applicable	12

The SPIRIT Explanation and Elaboration paper is distributed under the terms of the Creative Commons Attribution License CC-BY-NC. This checklist was completed on 10. October 2022 using https://www.goodreports.org/, a tool made by the EQUATOR Network in collaboration with Penelope.ai

For peer review only

BMJ Open

Effects of physical, psychosocial and dual-career loads on injuries and illnesses among elite handball players: protocol of prospective cohort study

Journal:	*BMJ Open*
Manuscript ID	bmjopen-2022-069104.R1
Article Type:	Protocol
Date Submitted by the Author:	31-Jan-2023
Complete List of Authors:	Drole, Kristina; University of Ljubljana<br>Paravlic, Armin; University of Ljubljana; Masaryk University<br>Steffen, Kathrin; Norwegian School of Sport Sciences<br>Doupona, Mojca; University of Ljubljana
**Primary Subject Heading**:	Sports and exercise medicine
Secondary Subject Heading:	Sports and exercise medicine
Keywords:	SPORTS MEDICINE, Health policy < HEALTH SERVICES ADMINISTRATION & MANAGEMENT, IMMUNOLOGY, Biochemistry < NATURAL SCIENCE DISCIPLINES

SCHOLARONE™
Manuscripts

Proposed journal: BMJ Open

Title: Effects of physical, psychosocial and dual-career loads on injuries and illnesses among elite handball players: protocol of prospective cohort study

Authors: Kristina Drole[1], Armin Paravlic[1,2,3], Kathrin Steffen[4], Mojca Doupona[1]

**Corresponding author:*

Kristina Drole
Faculty of Sport, University of Ljubljana
Gortanova 22, 1000 Ljubljana, Slovenia
E-mail: Kristina.drole@fsp.uni-lj.si

[1] Faculty of Sport, University of Ljubljana, Ljubljana, Slovenia
[2] Science and Research Centre Koper, Institute for Kinesiology Research, Koper, Slovenia
[3] Faculty of Sports Studies, Masaryk University, Brno, Czechia
[4] Oslo Sports Trauma Research Center, Norwegian School of Sport Sciences, Oslo, Norway

ORCID:
Kristina Drole: 0000-0002-8403-3154
Armin Paravlic: 0000-0002-7748-8097
Kathrin Steffen: 0000-0002-6238-608X
Mojca Doupona: 0000-0002-6715-0391

Word count: 2104

For peer review only

Abstract

Introduction

Health problems in sport cause a major burden on several pillars: sport clubs, health and insurance system and mostly the individual athlete. There is limited knowledge in supporting dual-career athletes based on evidence-based research in injury/illness prevention, load and stress management. The main goal of this research approach is to determine how specific physical, psychosocial and dual-career loads affect the occurrence of injuries and illnesses in elite handball players and how much of a variation in the athlete's load leads to an occurrence of an injury/illness. A secondary aim is to determine the association between objective and subjective measures of stress, as well examine the benefits of certain biomarkers to monitor stress, load and injury/illness occurrence in athletes.

Methods and analysis

This prospective cohort study, as part of a PhD-project, will be carried out on 200 elite handball players of 1st men handball league in Slovenia during an entire handball season, lasting from July 2022 to June 2023. Primary outcomes, including health problems, loads and stress, will be assessed weekly on a player level. Other player-related outcomes will include anthropometry, life event survey and blood biomarkers (cortisol, free testosterone and Ig-A), which will be taken three to five times across the observation period according to the players´ training cycle.

Ethics and dissemination

The project was approved by the National Medical Ethics Committee of Slovenia (number: 0120-109/2022/3) and will be conducted in compliance with the most recent version of Helsinki Declaration. The study results will be published as peer-reviewed articles, congress presentations and as a Doctoral thesis. The results will not only be of importance for the medical and sports community for development of new injury prevention and rehabilitation strategies, but also for structuring the correct policy recommendations for athletes' general health.

Registration details

Trial registration number: NCT0547129

Keywords: monitoring, athletic injuries, health problems, biomarkers, load management

For peer review only

For peer review only - http://bmjopen.bmj.com/site/about/guidelines.xhtml

Article Summary

Strengths and limitations of this study

- This longitudinal study will investigate the associations between different types of load and injury/illness occurrence in elite handball players
- The study will implement holistic monitoring of athlete's health with objective and subjective measures
- The association between objective and subjective measures of stress in athletes will be investigated
- The study focuses exclusively on elite male handball players, which can be seen both as a strength and also as a limitation when it comes to generalizability
- The work has direct implications for sports/clinical practice; periodical athlete monitoring could be beneficial in terms of preventing health issues, lowering health system costs, supporting dual career of athletes, structuring sound policies and enhancing both sports and academic performance

For peer review only

For peer review only - http://bmjopen.bmj.com/site/about/guidelines.xhtml

Introduction

Regular moderate exercise is known to have positive health benefits[1]; from preventing a number of non-communicable diseases to improving cardio-respiratory fitness and consequently decreasing cardiovascular morbidity and mortality.[1–3] Top-level sports on the other hand, are associated with an increased risk for illness and injury.[4,5] There has been a number of studies[6–12] showing a high prevalence of health issues that include injuries and illnesses among elite athletes, ranging from 20% in athletes of all ages[4] to 45% in youth athletes.[5] The injuries and illnesses are not only limiting athletes for the time being[13], but also represent a major burden for sport clubs, health and insurance system and can as well be the reason for sports career termination.[14–18]

Athlete's stressors

Sports and non-sports loads impose stress on athletes, leading their physical and psychological well-being from homeostasis to acute fatigue, over-reaching, overtraining, subclinical immune changes, clinical symptoms, illness/injury and possibly death.[19] Research points out that inadequate load management is an important independent risk factor for the development of acute illness and overtraining syndrome.[9,19,20] However, it is not yet possible to quantify how much an increase in training load leads to an increased risk for a given illness[19] or injury.[21] Furthermore, the literature states that there is a risk of illness when an athlete is exposed to other lifestyle stressors that affect the immune system, including sleep deprivation or severe psychosocial stress[22], such as major life events.[23] A special group of athletes, that might be at higher risk for health problems are dual-career (DC) athletes. They are posed to the challenge of combining their education/work with elite sport, suggesting their overall weekly load is higher compared to non-athlete students and athletes who are not in the education process. It was found that the highest levels of stress and illness incidence in student-athletes arise during the academically stressful periods.[24,25] Thus, the relationship between stress and physical health is especially relevant for dual-career athletes, who often face multiple physical and psychosocial stressors (such as transitioning between schools/campuses and home life, frequent travel, academic load and sports load).[26–28]

Health monitoring and load management

One potential strategy for injury and illness prevention in elite sport is monitoring of external and internal loads. Monitoring an athlete's workload has a number of potential benefits, including interpreting performance changes, increasing understanding of exercise responses, detecting fatigue and monitoring recovery needs, optimization of the training programme and competition calendar. It also serves for providing adequate workload levels to reduce the risk of injury and illness[29,30] and guide rehabilitation programmes. External and internal loads can be assessed with subjective and objective measures. External load measurement typically involves quantifying an athlete's training or competition loads by reporting training hours, running length, amount of weight lifted, number of games played and other external factors such as life events, everyday problems and/or travel. On the other hand, internal load is measured by assessing the internal biological, physiological, and psychological response to an external load[31], such as heart rate or ratings of perceived exertion. A recent systematic review of internal stress monitoring has identified subjective reporting to be more sensitive and consistent than objective measurements in determining acute and chronic changes in an athlete's well-being.[32]

For peer review only - http://bmjopen.bmj.com/site/about/guidelines.xhtml

Biomarkers

Biomarker discovery and validation is a critical aim of the medical and scientific community.[33] Biomarkers can be used as an objective assessment tool for health, performance, recovery and talent identification in elite sport. Among the most critical hormones for athletes seem to be cortisol, testosterone, dehydroepiandrosterone (DHEA) and immunoglobulin-A (Ig-A). However, T:C ratio is considered to be more sensitive to training stress than either measure alone[33], as several studies report poor performance outcomes and suboptimal training adaptations in athletes with a low T:C ratio.[34,35] However, to the best of the authors knowledge, no previous work has examined the use of biomarkers for injury prevention, recovery after an injury or identification of individuals at increased risk.

Given the all above, there is a need for better health surveillance, load monitoring and support services with an emphasis on injury/illness prevention and rehabilitation.[36] As DC athletes have a wider variety of loads that pose greater psychophysiological strain to them, they could also be at an increased risk for injury/illness. Therefore, more comprehensive monitoring of potential risk factors in this specific population is warranted. It is not yet known, but important to determine how much of overall load can an athlete withstand before injury/illness occurs. Thus, the primary aim of this study is to determine how specific physical, psychosocial and dual-career loads affect the occurrence of injuries and illnesses in elite handball players and how much of a variation in the athlete's load leads to an occurrence of an injury/illness. A secondary aim is to determine the association between objective and subjective measures of stress, as well as examine the benefits of certain biomarkers to monitor stress, load and injury/illness occurrence in athletes.

Methods and analysis

Study design

The study is designed as prospective cohort study. All measurements will be conducted over a single handball season, lasting from July 2022 to June 2023 (a total of 46 weeks), with weekly reporting (Table 1) and three to five biomarker sampling timepoints (Figure 1). We used the SPIRIT checklist when writing our report. [37]

Participants and sample size

A power analysis was a priory determined using the G*Power software.[38] Based on previous studies with similar design we expected to find nearly twice as high odds of injury occurrence in athletes during a period of high academic stress compared to low academic stress (odds ratio [OR] = 1.78; effect size [ES] = 0.14)[25] with power of 0.90 and α = 0.05, two-tailed, which calculated an initial sample size of 134 participants. As in sports practice, the athlete drop-out is to be expected (due to travelling, international transfer to another club/state etc.); we adjusted the originally calculated sample size by following formula: N1 = n/(1-d)[39], where N1 is adjusted sample size, n is the sample size required as per the proposed formula (N = 134), and d is the drop-out rate (d = 0.10). This resulted in total sample size of 149 athletes. Inclusion criteria will be as follows: DC and non-DC registered male handball players who are competing in the 1st Slovenian handball league and are above 18 years of age.

For peer review only - http://bmjopen.bmj.com/site/about/guidelines.xhtml

Assessment and Outcomes

*** Insert Figure 1 "Assessment plan" approximately here ***

Oslo Sports Trauma Research Centre Questionnaire on Health Problems (OSTRC-H2)

The OSTRC questionnaire is a tool to capture and monitor health problems longitudinally in athletes. It consists of 18 questions, which are answered for the past seven days. The first four questions are to determine the severity of the health problem, while the rest are to further classify the health problem, such as type and anatomical location of the problem.[40] As per previously established protocol, the athletes in our study will self-report their health status weekly with the help of a physiotherapist, while using the OSTRC questionnaire.[40] The athletes with reported health problem will be contacted by a medical professional to clinically verify the reported health case.

Life Events Survey for Collegiate Athletes Questionnaire (LESCA)

At the baseline and final measurements, the athletes will be asked to complete LESCA questionnaire[41] in order to get more contextualized view of the life events that were happening in the past 12 months. LESCA is a 69-item questionnaire where athletes rate how each life event affected their lives in the past 12 months on a -4 to 4-point scale (-4, extremely negative to +4, extremely positive). The events include personal, academic-related and sports-related events. From summing the rated life events, two life stress scores, negative (NEG) and positive (POS) are obtained, while a total life stress score can as well be obtained by summing the NEG and POS scores together.[41]

Weekly load and stress Questionnaire

The teams will weekly report training and competition load for each athlete, while academic (educational activities, including lectures, exams and studying) and work load (a part-time or full-time job besides playing sports) for the past seven days will be reported by the athletes themselves. Competition load will be reported in minutes for each game played in the past week, while training, academic and workload will be reported in hours. The athletes will additionally report average hours slept per day over the past week and rate their perceived overall stress of that week on the scale 1-10.

Blood biomarkers

There will be three to five measurement timepoints for biomarker sampling, according to the training (preparatory period, competitive period, off-season period) cycle. The athletes will be tested under similar conditions every time, after a rest day (refrainment from intensive training the day before blood collection), between 7-10 a.m.

Table 1. Questionnaires and tests.

Test/Questionnaire	Outcome	Purpose
**SDSCQ**	Age, sex, level of education, sport, years of training, player position, past injuries	General information about the athlete

For peer review only - http://bmjopen.bmj.com/site/about/guidelines.xhtml

**Anthropometry**	Body height (cm), body weight (kg)	Measure of basic anthropometric characteristics of an athlete
**LESCA**	Major life events score	Measure of life stress
**OSTRC**	Weekly health problems (acute and overuse injuries, illnesses), measured as prevalence and incidence and severity as OSTRC-severity score and time-loss days	Identifying health problems
**Weekly load and stress**		Monitoring of athlete's load
	Training load in hours	
	Competition load in minutes	
	Academic load in hours	
	Work load in hours	
	Average daily sleeping hours	
	Perceived stress on scale 1-10	
**Blood biomarkers**		Monitoring of stress/insufficient recovery and/or health problem risk
Cortisol	Cortisol values in nmol/L	
Free testosterone	Free testosterone values in pmol/L	
Ig-A	Ig-A values in g/L	
	Free testosterone: cortisol ratio	

SDSCQ, sociodemographic and sports-career related questionnaire; LESCA, Life Events Survey for Collegiate Athletes; OSTRC, Oslo Sports Trauma Research Center Questionnaire on Health Problems

Statistical analysis

Statistical analysis will be performed using standard statistical software (SPSS 27.0, IBM Corp, Armonk, NY, USA). Descriptive statistics will be used for all variables. Normality of data distribution will be assessed using the Shapiro-Wilk test. In case of non-normally distributed data, non-parametric statistics will be used, otherwise, the statistics for normally distributed data will be utilized. The data will be analysed using a logistic regression model to properly account for the dependency induced by the repeated observations over time within each subject. Main effect of all dependent variables on injury/illness occurrence will be studied using repeated measures ANOVA with time (preparation period vs. competitive period vs. off-season period) as within factor, while group (Injured/ill vs. Healthy) as between factor. Where significant effects will be found for group, time effects or 2-way interactions, pairwise comparisons will be used to address significant differences for each variable independently. For comparison of the groups we will use independent t-test. The magnitude of effect for all dependent variables will be reported as partial eta squared (η^2). In addition, *Cohens' d* (ES) will be used to assess magnitude of difference between groups. Furthermore, regression analysis will be used to assess predictive power of individual parameters to identify injury/illness occurrence. For the correlation of subjective measures of stress with the objective measures, bivariate correlation will be used. Additionally, Bland-Altmann analysis will be used (low/high stress normative values) as previously recommended.[42] Furthermore, ROC curve will be used for defining the cut-off values. Hypotheses will be accepted or rejected at $P \leq 0.05$.

For peer review only - http://bmjopen.bmj.com/site/about/guidelines.xhtml

Patient and public involvement

Patients and/or the public were not involved in the design and/or conduct, and/or reporting of this study. The project results will be presented to the participants in collaboration with the team staff (strength and conditioning coaches, physiotherapists, kinesiologists, etc.). Furthermore, the participants of the proposed study will be invited to share their experiences of their involvement in our project with elite athletes playing other sports in the Republic of Slovenia in order to continue to implement "good practice" with future projects.

Ethics and dissemination

The project was approved by the National Medical Ethics Committee of Slovenia (number: 0120-109/2022/3) and registered on Clinical Trials.gov (registration number: NCT05471297). The study will be conducted in compliance with the most recent version of Helsinki Declaration of 1975 and the American College of Sports Medicine Policy Statement Regarding the Use of Human Subjects and Informed Consent. Any changes made to the study protocol will be submitted to the National Medical Ethics Committee for consideration and approval. Participants will be introduced to the aims and objectives of the study, noticed that participation in the study is voluntary and asked to sign an informed consent, collected by KD. Consent document also states that the participant is free to withdraw from the study without impact on their future status in the team and without having to provide the reason for their withdrawal. Data on each participant will be anonymised using only ID numbers. The study will be coordinated by KD, a Ph.D. student conducting her Ph.D. thesis on this topic. As such, the study results will be published as peer-reviewed articles, conference presentations and finally, as a Doctoral thesis.

Acknowledgements

The authors wish to thank University Medical Centre Ljubljana, Clinical Institute of Clinical Chemistry and Biochemistry for supporting the upcoming blood biomarker analysis.

Author contributions

KD contributed to the idea, conceptualization and drafted the manuscript; KD and AP contributed to design and planned analysis of the study. AP, KS and MD critically revised the manuscript. KD, AP, KS and MD gave their final approval for submission.

Funding

The work is supported by a research fellowship grant (no. 393/2020) received by KD from the Public Research Agency of the Republic of Slovenia (ARRS). The research took place within the KINESIOLOGY OF MONOSTRUCTURAL, POLYSTRUCTURAL AND CONVENTIONAL SPORTS research program, code: P5-0147, financed by the Public Research Agency of the Republic of Slovenia. The funding agency has no impact on data collection, analysis or interpretation of the study results.

Competing interests

The authors report no competing interest within the submitted work.

For peer review only - http://bmjopen.bmj.com/site/about/guidelines.xhtml

Data statement

Data will be provided by the corresponding author upon a reasonable request.

References

1 Ruegsegger GN, Booth FW. Health Benefits of Exercise. *Cold Spring Harb Perspect Med* 2018;**8**. doi:10.1101/cshperspect.a029694

2 Warburton DER, Nicol CW, Bredin SSD. Health benefits of physical activity: the evidence. *C Can Med Assoc J = J l'Association medicale Can* 2006;**174**:801–9. doi:10.1503/cmaj.051351

3 Warburton DER, Bredin SSD. Health benefits of physical activity: a systematic review of current systematic reviews. *Curr Opin Cardiol* 2017;**32**:541–56. doi:10.1097/HCO.0000000000000437

4 Pluim BM, Fuller CW, Batt ME, *et al.* Consensus statement on epidemiological studies of medical conditions in tennis, April 2009. *Clin J Sport Med Off J Can Acad Sport Med* 2009;**19**:445–50. doi:10.1097/JSM.0b013e3181be35e5

5 Moseid CH, Myklebust G, Fagerland MW, *et al.* The prevalence and severity of health problems in youth elite sports: A 6-month prospective cohort study of 320 athletes. *Scand J Med Sci Sports* 2018;**28**:1412–23. doi:10.1111/sms.13047

6 Steffen K, Moseid CH, Engebretsen L, *et al.* Sports injuries and illnesses in the Lillehammer 2016 Youth Olympic Winter Games. *Br J Sports Med* 2017;**51**:29–35. doi:10.1136/bjsports-2016-096977

7 Steffen K, Soligard T, Mountjoy M, *et al.* How do the new Olympic sports compare with the traditional Olympic sports? Injury and illness at the 2018 Youth Olympic Summer Games in Buenos Aires, Argentina. *Br J Sports Med* 2020;**54**:168–75. doi:10.1136/bjsports-2019-101040

8 Engebretsen L, Soligard T, Steffen K, *et al.* Sports injuries and illnesses during the London Summer Olympic Games 2012. *Br J Sports Med* 2013;**47**:407–14.

9 Soligard T, Steffen K, Palmer-Green D, *et al.* Sports injuries and illnesses in the Sochi 2014 Olympic Winter Games. *Br J Sports Med* 2015;**49**:441–7. doi:10.1136/bjsports-2014-094538

10 Steffen K, Clarsen B, Gjelsvik H, *et al.* Illness and injury among Norwegian Para athletes over five consecutive Paralympic Summer and Winter Games cycles: prevailing high illness burden on the road from 2012 to 2020. *Br J Sports Med* 2022;**56**:204–12. doi:10.1136/bjsports-2021-104489

11 Theron N, Schwellnus M, Derman W, *et al.* Illness and injuries in elite football players--a prospective cohort study during the FIFA Confederations Cup 2009. *Clin J Sport Med Off J Can Acad Sport Med* 2013;**23**:379–83. doi:10.1097/JSM.0b013e31828b0a10

12 Rosen P von, Heijne A, Frohm A, *et al.* High Injury Burden in Elite Adolescent Athletes: A 52-Week Prospective Study. *J Athl Train (Allen Press* 2018;**53**:262–70.

13 Caine DJ, Golightly YM. Osteoarthritis as an outcome of paediatric sport: an epidemiological perspective. *Br J Sports Med* 2011;**45**:298–303. doi:10.1136/bjsm.2010.081984

14 Drawer S, Fuller CW. Propensity for osteoarthritis and lower limb joint pain in retired professional soccer players. *Br J Sports Med* 2001;**35**:402–8. doi:10.1136/bjsm.35.6.402

15 Palmer D, Cooper DJ, Emery C, *et al.* Self-reported sports injuries and later-life health status in 3357 retired Olympians from 131 countries: A cross-sectional survey among those competing in the games between London 1948 and PyeongChang 2018. *Br J Sports Med* 2021;**55**:46–53.

For peer review only - http://bmjopen.bmj.com/site/about/guidelines.xhtml

doi:10.1136/bjsports-2019-101772

16 Drole K. *ZDRAVSTVENI STATUS IN POŠKODBE BIVŠIH VRHUNSKIH ŠPORTNIKOV*. 2020.

17 Leena R, Kettunen J, Kujala U, *et al.* Sport injuries as the main cause of sport career termination among Finnish top-level athletes. *Eur J Sport Sci* 2012;**12**:274–82. doi:10.1080/17461391.2011.566365

18 Kettunen JA, Kujala UM, Kaprio J, *et al.* Lower-limb function among former elite male athletes. *Am J Sports Med* 2001;**29**:2–8. doi:10.1177/03635465010290010801

19 Schwellnus M, Soligard T, Alonso J-M, *et al.* How much is too much? (Part 2) International Olympic Committee consensus statement on load in sport and risk of illness. *Br J Sports Med* 2016;**50**:1043–52. doi:10.1136/bjsports-2016-096572

20 Gleeson M, Pyne DB. Respiratory inflammation and infections in high-performance athletes. *Immunol Cell Biol* 2016;**94**:124–31. doi:10.1038/icb.2015.100

21 Dalen-Lorentsen T. *Training load and health problems in football –More complex than we first thought?* 2021.

22 NIEMAN DC. Exercise effects on systemic immunity. *Immunol Cell Biol* 2000;**78**:496–501. doi:https://doi.org/10.1111/j.1440-1711.2000.t01-5-.x

23 Smith RE, Smoll FL, Ptacek JT. Conjunctive moderator variables in vulnerability and resiliency research: Life stress, social support and coping skills, and adolescent sport injuries. J. Pers. Soc. Psychol. 1990;**58**:360–70. doi:10.1037/0022-3514.58.2.360

24 Hamlin MJ, Wilkes D, Elliot CA, *et al.* Monitoring Training Loads and Perceived Stress in Young Elite University Athletes. *Front Physiol* 2019;**10**:34. doi:10.3389/fphys.2019.00034

25 Mann JB, Bryant KR, Johnstone B, *et al.* Effect of Physical and Academic Stress on Illness and Injury in Division 1 College Football Players. *J strength Cond Res* 2016;**30**:20–5. doi:10.1519/JSC.0000000000001055

26 Condello G, Capranica L, Doupona M, *et al.* Dual-career through the elite university student-athletes' lenses: The international FISU-EAS survey. *PLoS One* 2019;**14**:e0223278.https://doi.org/10.1371/journal.pone.0223278

27 Fuchs PX, Doupona M, Varga K, *et al.* Multi-national perceptions on challenges, opportunities, and support structures for Dual Career migrations in European student-athletes. *PLoS One* 2021;**16**:e0253333. doi:10.1371/journal.pone.0253333

28 Cosh SM, Tully PJ. Stressors, coping, and support mechanisms for student athletes combining elite sport and tertiary education: implications for practice. *IEEE Trans Signal Process* 2015;**29**:120–33.

29 Halson SL. Monitoring training load to understand fatigue in athletes. *Sports Med* 2014;**44 Suppl 2**:S139-47. doi:10.1007/s40279-014-0253-z

30 Foster C. Monitoring training in athletes with reference to overtraining syndrome. *Med Sci Sports Exerc* 1998;**30**:1164–8. doi:10.1097/00005768-199807000-00023

31 Borresen J, Lambert MI. The quantification of training load, the training response and the effect on performance. *Sports Med* 2009;**39**:779–95. doi:10.2165/11317780-000000000-00000

32 Saw AE, Main LC, Gastin PB. Monitoring the athlete training response: Subjective self-reported measures trump commonly used objective measures: A systematic review. *Br J Sports Med* 2016;**50**:281–91. doi:10.1136/bjsports-2015-094758

For peer review only - http://bmjopen.bmj.com/site/about/guidelines.xhtml

33 Lee EC, Fragala MS, Kavouras SA, *et al.* Biomarkers in Sports and Exercise: Tracking Health, Performance, and Recovery in Athletes. *J strength Cond Res* 2017;**31**:2920–37. doi:10.1519/JSC.0000000000002122

34 Kraemer WJ, French DN, Paxton NJ, *et al.* Changes in exercise performance and hormonal concentrations over a big ten soccer season in starters and nonstarters. *J strength Cond Res* 2004;**18**:121–8. doi:10.1519/1533-4287(2004)018<0121:ciepah>2.0.co;2

35 Chicharro JL, López-Mojares LM, Lucía A, *et al.* Overtraining parameters in special military units. *Aviat Space Environ Med* 1998;**69**:562–8.

36 Drole K, Paravlic AH. Interventions for increasing return to sport rates after an anterior cruciate ligament reconstruction surgery: A systematic review. Front. Psychol. 2022;**13**:939209. doi:10.3389/fpsyg.2022.939209

37 Chan A-W, Tetzlaff JM, Gøtzsche PC, *et al.* SPIRIT 2013 explanation and elaboration: guidance for protocols of clinical trials. *BMJ* 2013;**346**:e7586. doi:10.1136/bmj.e7586

38 Faul F, Erdfelder E, Lang A-G, *et al.* G*Power 3: a flexible statistical power analysis program for the social, behavioral, and biomedical sciences. *Behav Res Methods* 2007;**39**:175–91. doi:10.3758/bf03193146

39 Sakpal TV. Sample size estimation in clinical trial. *Perspect Clin Res* 2010;**1**:67–9.

40 Clarsen B, Bahr R, Myklebust G, *et al.* Improved reporting of overuse injuries and health problems in sport: an update of the Oslo Sport Trauma Research Center questionnaires. *Br J Sports Med* 2020;**54**:390–6. doi:10.1136/bjsports-2019-101337

41 Petrie TA. Psychosocial antecedents of athletic injury: the effects of life stress and social support on female collegiate gymnasts. *Behav Med* 1992;**18**:127–38. doi:10.1080/08964289.1992.9936963

42 Ludbrook J. Confidence in Altman-Bland plots: a critical review of the method of differences. *Clin Exp Pharmacol Physiol* 2010;**37**:143–9. doi:10.1111/j.1440-1681.2009.05288.x

For peer review only

For peer review only - http://bmjopen.bmj.com/site/about/guidelines.xhtml

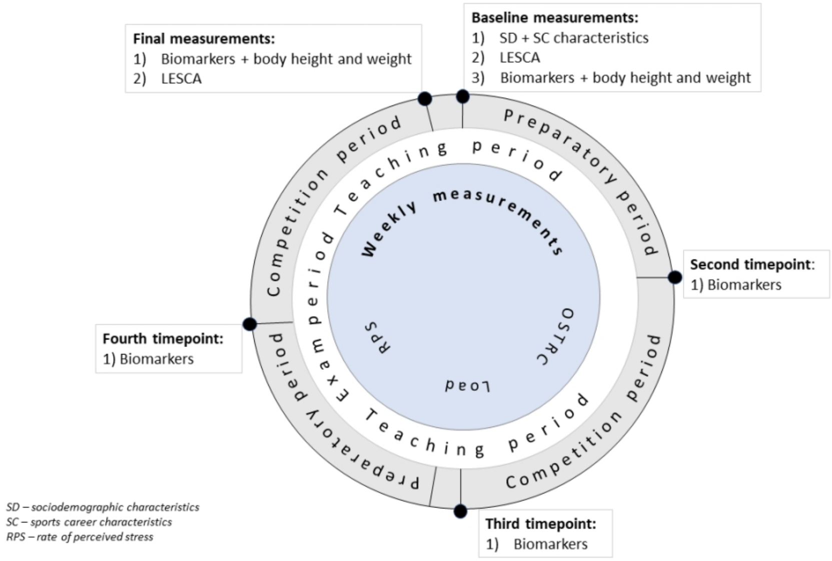

338x190mm (96 x 96 DPI)

Reporting checklist for protocol of a clinical trial.

Based on the SPIRIT guidelines.

Instructions to authors

Complete this checklist by entering the page numbers from your manuscript where readers will find each of the items listed below.

Your article may not currently address all the items on the checklist. Please modify your text to include the missing information. If you are certain that an item does not apply, please write "n/a" and provide a short explanation.

Upload your completed checklist as an extra file when you submit to a journal.

In your methods section, say that you used the SPIRITreporting guidelines, and cite them as:

Chan A-W, Tetzlaff JM, Gøtzsche PC, Altman DG, Mann H, Berlin J, Dickersin K, Hróbjartsson A, Schulz KF, Parulekar WR, Krleža-Jerić K, Laupacis A, Moher D. SPIRIT 2013 Explanation and Elaboration: Guidance for protocols of clinical trials. BMJ. 2013;346:e7586

		Reporting Item	Page Number
**Administrative information**			
Title	#1	Descriptive title identifying the study design, population, interventions, and, if applicable, trial acronym	1
Trial registration	#2a	Trial identifier and registry name. If not yet registered, name of intended registry	2
Trial registration: data set	#2b	All items from the World Health Organization Trial Registration Data Set	2
Protocol version	#3	Date and version identifier	2
Funding	#4	Sources and types of financial, material, and other support	8
Roles and responsibilities: contributorship	#5a	Names, affiliations, and roles of protocol contributors	1

For peer review only

Roles and responsibilities: sponsor contact information	#5b	Name and contact information for the trial sponsor	NA
Roles and responsibilities: sponsor and funder	#5c	Role of study sponsor and funders, if any, in study design; collection, management, analysis, and interpretation of data; writing of the report; and the decision to submit the report for publication, including whether they will have ultimate authority over any of these activities	8
Roles and responsibilities: committees	#5d	Composition, roles, and responsibilities of the coordinating centre, steering committee, endpoint adjudication committee, data management team, and other individuals or groups overseeing the trial, if applicable (see Item 21a for data monitoring committee)	NA
**Introduction**			
Background and rationale	#6a	Description of research question and justification for undertaking the trial, including summary of relevant studies (published and unpublished) examining benefits and harms for each intervention	4-5
Background and rationale: choice of comparators	#6b	Explanation for choice of comparators	NA
Objectives	#7	Specific objectives or hypotheses	5
Trial design	#8	Description of trial design including type of trial (eg, parallel group, crossover, factorial, single group), allocation ratio, and framework (eg, superiority, equivalence, non-inferiority, exploratory)	5
**Methods: Participants, interventions, and outcomes**			
Study setting	#9	Description of study settings (eg, community clinic, academic hospital) and list of countries where data will be collected. Reference to where list of study sites can be obtained	5
Eligibility criteria	#10	Inclusion and exclusion criteria for participants. If applicable, eligibility criteria for study centres and individuals who will	5

For peer review only

		perform the interventions (eg, surgeons, psychotherapists)	
Interventions: description	#11a	Interventions for each group with sufficient detail to allow replication, including how and when they will be administered	5-7
Interventions: modifications	#11b	Criteria for discontinuing or modifying allocated interventions for a given trial participant (eg, drug dose change in response to harms, participant request, or improving / worsening disease)	5
Interventions: adherance	#11c	Strategies to improve adherence to intervention protocols, and any procedures for monitoring adherence (eg, drug tablet return; laboratory tests)	NA
Interventions: concomitant care	#11d	Relevant concomitant care and interventions that are permitted or prohibited during the trial	6
Outcomes	#12	Primary, secondary, and other outcomes, including the specific measurement variable (eg, systolic blood pressure), analysis metric (eg, change from baseline, final value, time to event), method of aggregation (eg, median, proportion), and time point for each outcome. Explanation of the clinical relevance of chosen efficacy and harm outcomes is strongly recommended	6-7
Participant timeline	#13	Time schedule of enrolment, interventions (including any run-ins and washouts), assessments, and visits for participants. A schematic diagram is highly recommended (see Figure)	6
Sample size	#14	Estimated number of participants needed to achieve study objectives and how it was determined, including clinical and statistical assumptions supporting any sample size calculations	5
Recruitment	#15	Strategies for achieving adequate participant enrolment to reach target sample size	5
**Methods: Assignment of interventions (for controlled trials)**			
Allocation: sequence generation	#16a	Method of generating the allocation sequence (eg, computer-generated random numbers), and list of any factors for stratification. To reduce predictability of a random sequence, details of any planned restriction (eg, blocking) should be provided in a separate document that is unavailable to those who enrol participants or assign interventions	NA

For peer review only

Allocation concealment mechanism	#16b	Mechanism of implementing the allocation sequence (eg, central telephone; sequentially numbered, opaque, sealed envelopes), describing any steps to conceal the sequence until interventions are assigned	NA
Allocation: implementation	#16c	Who will generate the allocation sequence, who will enrol participants, and who will assign participants to interventions	NA
Blinding (masking)	#17a	Who will be blinded after assignment to interventions (eg, trial participants, care providers, outcome assessors, data analysts), and how	NA
Blinding (masking): emergency unblinding	#17b	If blinded, circumstances under which unblinding is permissible, and procedure for revealing a participant's allocated intervention during the trial	NA
**Methods: Data collection, management, and analysis**			
Data collection plan	#18a	Plans for assessment and collection of outcome, baseline, and other trial data, including any related processes to promote data quality (eg, duplicate measurements, training of assessors) and a description of study instruments (eg, questionnaires, laboratory tests) along with their reliability and validity, if known. Reference to where data collection forms can be found, if not in the protocol	5-7
Data collection plan: retention	#18b	Plans to promote participant retention and complete follow-up, including list of any outcome data to be collected for participants who discontinue or deviate from intervention protocols	5-7
Data management	#19	Plans for data entry, coding, security, and storage, including any related processes to promote data quality (eg, double data entry; range checks for data values). Reference to where details of data management procedures can be found, if not in the protocol	7-8
Statistics: outcomes	#20a	Statistical methods for analysing primary and secondary outcomes. Reference to where other details of the statistical analysis plan can be found, if not in the protocol	7
Statistics: additional analyses	#20b	Methods for any additional analyses (eg, subgroup and adjusted analyses)	7

For peer review only

Statistics: analysis population and missing data	#20c	Definition of analysis population relating to protocol non-adherence (eg, as randomised analysis), and any statistical methods to handle missing data (eg, multiple imputation)	7
**Methods: Monitoring**			
Data monitoring: formal committee	#21a	Composition of data monitoring committee (DMC); summary of its role and reporting structure; statement of whether it is independent from the sponsor and competing interests; and reference to where further details about its charter can be found, if not in the protocol. Alternatively, an explanation of why a DMC is not needed	NA
Data monitoring: interim analysis	#21b	Description of any interim analyses and stopping guidelines, including who will have access to these interim results and make the final decision to terminate the trial	NA
Harms	#22	Plans for collecting, assessing, reporting, and managing solicited and spontaneously reported adverse events and other unintended effects of trial interventions or trial conduct	NA
Auditing	#23	Frequency and procedures for auditing trial conduct, if any, and whether the process will be independent from investigators and the sponsor	NA
**Ethics and dissemination**			
Research ethics approval	#24	Plans for seeking research ethics committee / institutional review board (REC / IRB) approval	7-8
Protocol amendments	#25	Plans for communicating important protocol modifications (eg, changes to eligibility criteria, outcomes, analyses) to relevant parties (eg, investigators, REC / IRBs, trial participants, trial registries, journals, regulators)	7-8
Consent or assent	#26a	Who will obtain informed consent or assent from potential trial participants or authorised surrogates, and how (see Item 32)	7-8
Consent or assent: ancillary studies	#26b	Additional consent provisions for collection and use of participant data and biological specimens in ancillary studies, if applicable	NA
Confidentiality	#27	How personal information about potential and enrolled participants will be collected, shared, and maintained in order to protect confidentiality before, during, and after the trial	7-8

For peer review only

Declaration of interests	#28	Financial and other competing interests for principal investigators for the overall trial and each study site	8
Data access	#29	Statement of who will have access to the final trial dataset, and disclosure of contractual agreements that limit such access for investigators	8
Ancillary and post trial care	#30	Provisions, if any, for ancillary and post-trial care, and for compensation to those who suffer harm from trial participation	NA
Dissemination policy: trial results	#31a	Plans for investigators and sponsor to communicate trial results to participants, healthcare professionals, the public, and other relevant groups (eg, via publication, reporting in results databases, or other data sharing arrangements), including any publication restrictions	7-8
Dissemination policy: authorship	#31b	Authorship eligibility guidelines and any intended use of professional writers	8
Dissemination policy: reproducible research	#31c	Plans, if any, for granting public access to the full protocol, participant-level dataset, and statistical code	8
**Appendices**			
Informed consent materials	#32	Model consent form and other related documentation given to participants and authorised surrogates	11
Biological specimens	#33	Plans for collection, laboratory evaluation, and storage of biological specimens for genetic or molecular analysis in the current trial and for future use in ancillary studies, if applicable	12

The SPIRIT Explanation and Elaboration paper is distributed under the terms of the Creative Commons Attribution License CC-BY-NC. This checklist was completed on 10. October 2022 using https://www.goodreports.org/, a tool made by the EQUATOR Network in collaboration with Penelope.ai

For peer review only
